# The Molecular Detection of Class B and Class D Carbapenemases in Clinical Strains of *Acinetobacter calcoaceticus-baumannii* Complex: The High Burden of Antibiotic Resistance and the Co-Existence of Carbapenemase Genes

**DOI:** 10.3390/antibiotics11091168

**Published:** 2022-08-30

**Authors:** Hasan Ejaz, Muhammad Usman Qamar, Kashaf Junaid, Sonia Younas, Zeeshan Taj, Syed Nasir Abbas Bukhari, Abualgasim E. Abdalla, Khalid O. A. Abosalif, Naveed Ahmad, Zikria Saleem, Eman H. M. Salem

**Affiliations:** 1 Department of Clinical Laboratory Sciences, College of Applied Medical Sciences, Jouf University, Sakaka 72388, Saudi Arabia; 2 Department of Microbiology, Faculty of Life Sciences, Government College University Faisalabad, Faisalabad 38000, Pakistan; 3 HKU-Pasteur Research Pole, School of Public Health, LKS Faculty of Medicine, The University of Hong Kong, Hong Kong, China; 4 Department of Pharmaceutical Chemistry, College of Pharmacy, Jouf University, Sakaka 72388, Saudi Arabia; 5 Department of Pharmaceutics, College of Pharmacy, Jouf University, Sakaka 72388, Saudi Arabia; 6 Department of Pharmacy Practice, Faculty of Pharmacy, Bahauddin Zakariya University, Multan 60000, Pakistan; 7 Department of Medical Microbiology and Immunology, Faculty of Medicine, Menoufia University, Shebin El-Koom 32511, Egypt; 8 Department of Microbiology, King AbdulAziz Specialist Hospital, Sakaka 72341, Saudi Arabia

**Keywords:** *Acinetobacter calcoaceticus-baumannii*, β-lactamase, carbapenemase, β-lactams, *bla*_NDM_, *bla*_OXA_, *bla*_VIM_

## Abstract

The emergence of carbapenem-resistant *Acinetobacter calcoaceticus*-*baumannii* complex (CRACB) in clinical environments is a significant global concern. These critical pathogens have shown resistance to a broad spectrum of antibacterial drugs, including carbapenems, mostly due to the acquisition of various β-lactamase genes. Clinical samples (n = 1985) were collected aseptically from multiple sources and grown on blood and MacConkey agar. Isolates and antimicrobial susceptibility were confirmed with the VITEK-2 system. The modified Hodge test confirmed the CRACB phenotype, and specific PCR primers were used for the molecular identification of *bla*_OXA_ and *bla*_NDM_ genes. Of the 1985 samples, 1250 (62.9%) were culture-positive and 200 (43.9%) were CRACB isolates. Of these isolates, 35.4% were recovered from pus samples and 23.5% from tracheal secretions obtained from patients in intensive care units (49.3%) and medical wards (20.2%). An antibiogram indicated that 100% of the CRACB isolates were resistant to β-lactam antibiotics and β-lactam inhibitors, 86.5% to ciprofloxacin, and 83.5% to amikacin, while the most effective antibiotics were tigecycline and colistin. The CRACB isolates displayed resistance to eight different AWaRe classes of antibiotics. All isolates exhibited the *bla*_OXA-51_ gene, while *bla*_OXA-23_ was present in 94.5%, *bla*_VIM_ in 37%, and *bla*_NDM_ in 14% of the isolates. The *bla*_OXA-51_, *bla*_OXA-23_, and *bla*_OXA-24_ genes co-existed in 13 (6.5%) isolates. CRACB isolates with co-existing *bla*_OXA-23_, *bla*_OXA-24_, *bla*_NDM_, *bla*_OXA-51_ and *bla*_VIM_ genes were highly prevalent in clinical samples from Pakistan. CRACB strains were highly critical pathogens and presented resistance to virtually all antibacterial drugs, except tigecycline and colistin.

## 1. Introduction

Carbapenem-resistant *Acinetobacter calcoaceticus*-*baumannii* complex (CRACB) is a well-known nosocomial pathogen that causes severe public health problems, primarily in low- and middle-income countries (LMICs) [1,2]. Recent data indicate that antimicrobial resistance (AMR) is becoming the leading cause of death; bacterial AMR was associated with 4.95 million deaths worldwide in 2019, of which 1.27 million deaths were caused by AMR, with relatively higher mortality among LMICs due to their fragile healthcare systems [3]. During the COVID-19 rife, excessive antimicrobial use resulted in an increase in AMR, which continues to pose a significant threat to healthcare [4]. *A. baumannii* is an important member of the non-Enterobacterales; this well-known pathogen is widespread and has a remarkable ability to develop AMR and cause persistent infections of hospital origin. It is responsible for high death rates ranging from 18.3% to 88.7%, depending on the source of infection [5]. As a result, the World Health Organization (WHO) has elevated it to the top of the list of critical pathogens. The Center for Disease Control and Prevention (CDC) placed it in the ESKAPE (*Enterococcus faecium*, *Staphylococcus aureus*, *Klebsiella pneumoniae*, *Acinetobacter baumannii*, *Pseudomonas aeruginosa*, and *Enterobacter* spp.) pathogens list [6]. Recent US data show that CRACB caused an estimated 700 deaths in 2017, and has contributed USD 281 million to health care costs annually [7].

Although carbapenems are the most powerful agents in the antibiotic armamentarium, the production of carbapenemases by bacteria, such as the CRACB strains that produce New Delhi metallo-β-lactamase (NDM) and oxacillinases (OXA), has immensely endangered the bactericidal properties of these medicines [8,9]. *A. baumannii*, which produces NDM and OXA, is resistant to various antibiotics and is only treated with antibiotics, such as colistin and polymyxin B, which are on the WHO’s critically important list of antimicrobials [10]. NDM was initially isolated from *Escherichia coli* and *Klebsiella pneumoniae* in 2009 from a Swedish patient admitted to a hospital in New Delhi, India [11]. In addition to carbapenem resistance mediated by oxacillinases, carbapenem activity is also impaired by metallo-β-lactamases (MBL), in particular by subclass B1 (NDM, VIM, and IMP) [12].

Depending on the amino acid sequence and substrate specificity, β-lactamases are divided into four Ambler’s classes: class A (penicillinases); class B (metallo-β-lactamases); class C (cephalosporinases); and class D (oxacillinases). The *bla*_NDM_ gene encodes a metallo-β-lactamase in subfamily B1 of class B and *bla*_OXA_ a class D β-lactamase [13]. Both of these β-lactamases have the highest number of genetic variants out of a total of 7420 β-lactamases (class B: 799; class D: 1142) [14]. Intrinsic *bla*_OXA-51_ is a chimeric determinant of *A. baumannii*, whereas *bla*_OXA-23_ and *bla*_OXA-24_ are acquired from *Acinetobacter radioresistens*. The continued increase in the number of these determinants due to unregulated diffusion is worrisome, as it challenges the effectiveness of antimicrobial agents, especially carbapenems [12]. Therefore, the present study highlights the acquisition, co-existence, and spread of these genetic determinants and challenges to infection control programs in hospitals.

## 2. Results

### 2.1. Prevalence of CRACB in the Clinical Setting

Of the 1985 clinical samples, 735 (37%) were bacterial culture-negative, and 1250 (62.9%) were culture-positive. Of the positive cultures, 795 (63.6%) isolates belonged to the Enterobacterales family and were excluded from the study, and the remaining 200 (43.9%) CRACB isolates were processed. Of the CRACB isolates, 79 (35.4%) were recovered from pus samples, 47 (23.5%) from tracheal secretions, 21 (10.5%) from sputum, and 20 (10%) from blood samples. The age range was 1 to 95 years, and the mean age was 43.5 years. Most of the isolates were recovered from males, and the male-to-female ratio was 1.27:1. The clinical samples were mainly collected from intensive care units (ICUs; 76 [38%]), followed by male medical wards (MMWs; 37 [18.5%]), female medical wards (FMWs; 36 [18%]), and critical care units (CCUs; 30 [15%]). There was no statistically significant association between the frequency of CRACB and gender, clinical samples, or ward; however, there was a significant association with age (*p* = 0.05) (Table 1).

### 2.2. Minimum Inhibitory Concentration of Antibiotics against CRACB 

**Figure 1:**
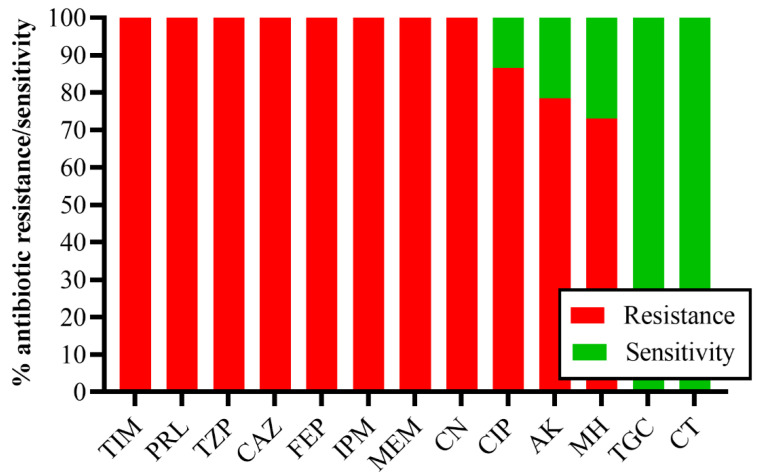
Antibiotic resistance profile of CRACB isolates. The different classes of antibiotics are TIM: ticarcillin/clavulanate; PRL: piperacillin; TZP: piperacillin/tazobactam; CAZ: ceftazidime; FEP: cefepime; IPM: imipenem; MEM: meropenem; CN: gentamicin; CIP: ciprofloxacin; AK: amikacin; MH: minocycline; TGC: tigecycline; CT: colistin.

**Figure 2:**
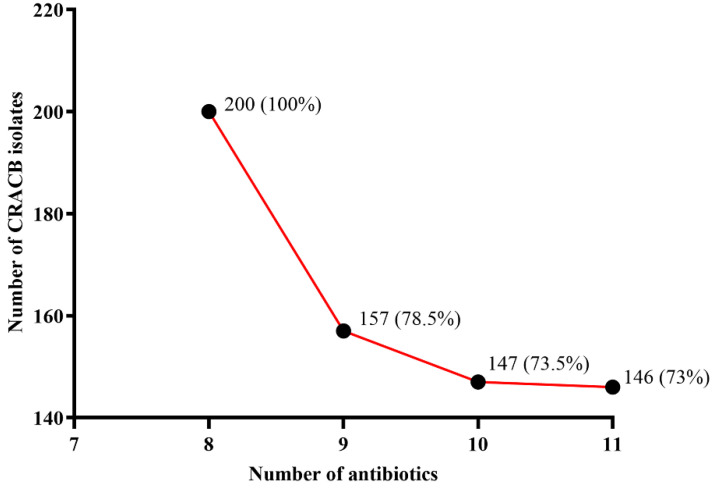
CRACB isolates concurrently resistant to the WHO AWaRe group of antibiotics. All the isolates were resistant to eight antibiotics.

Antimicrobial susceptibility testing revealed that all (100%) CRACB strains were resistant to all β-lactam antibiotics (cephalosporins and carbapenems) and β-lactamase inhibitors (ticarcillin/clavulanate and piperacillin/tazobactam), 86.5% to quinolone (ciprofloxacin), and 83.5% to aminoglycoside (amikacin) and tetracycline (minocycline), while the most effective antibiotics against CRACB were tigecycline and colistin (Figure 1). The WHO Expert Committee on the Selection and Use of Essential Medicines classified antibiotics into three categories: access, watch, and reserve (AWaRe). In this study, all (100%) CRACB displayed resistance to 8 different AWaRe antibiotics, 157 (78.5%) to 9 AWaRe antibiotics, 147 (73.5%) to 10 AWaRe antibiotics, and 146 (73%) to 11 AWaRe antibiotics (Figure 2).

### 2.3. Detection of Carbapenemases

**Figure 3:**
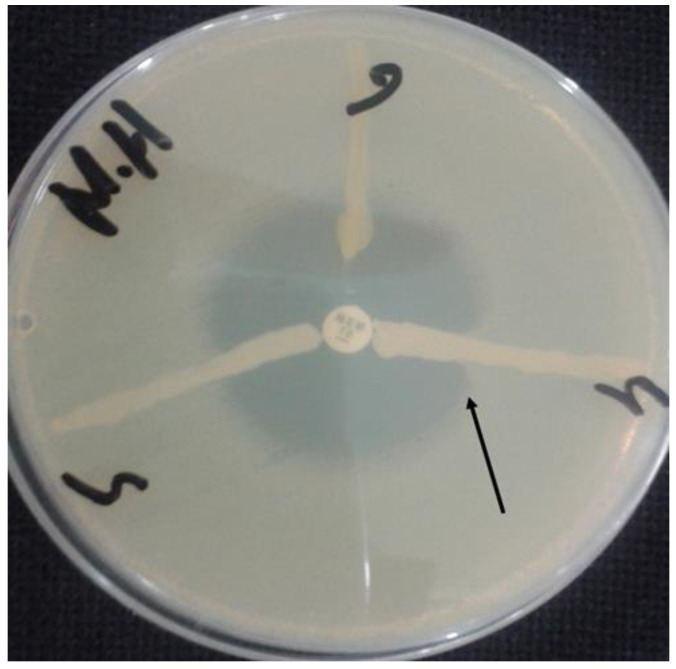
Phenotypic carbapenemase confirmation by the modified Hodge test.

Of the 200 CRACB isolates, 178 (89%) were positive for carbapenemase production, as shown by the modified Hodge’s test (Figure 3). The sources of the 178 carbapenemase-producing *Acinetobacter calcoaceticus-baumannii* complex (ACB) were as follows: 74 (41.5%) were from pus; 42 (23.6%) from tracheal secretions; 18 (10.1%) from sputum; 17 (9.5%) from blood; 15 (8.4%) from CSF; 8 (4.5%) from urine; and 4 (2.2%) from bile fluid.

### 2.4. Clinical Information on CRACB

We found that CRACB was disseminated in different hospital wards and could be isolated from various clinical sources. CRACB was mainly recovered from pus samples (79; 39.5%) followed by tracheal secretions (47; 23.5%), sputum (21; 10.5%), blood (20; 10%) and CSF (17; 8.5%). Most CRACB isolates from pus samples were detected in ICUs (31; 49.3%) and MMWs (16; 20.2%), while isolates from tracheal secretions were mainly collected from ICUs (19; 40.4%) and FMWs (11; 23.4%) and those from sputum and blood samples were obtained from MMWs (7; 33.3%) and ICUs (12; 60%), respectively. However, isolates from CSF and urine samples were mainly collected from FMWs (8; 47%) and MMWs (4; 36.6%), respectively (Table 2).

### 2.5. Genetic Determinants of Carbapenemase in CRACB

**Figure 4:**
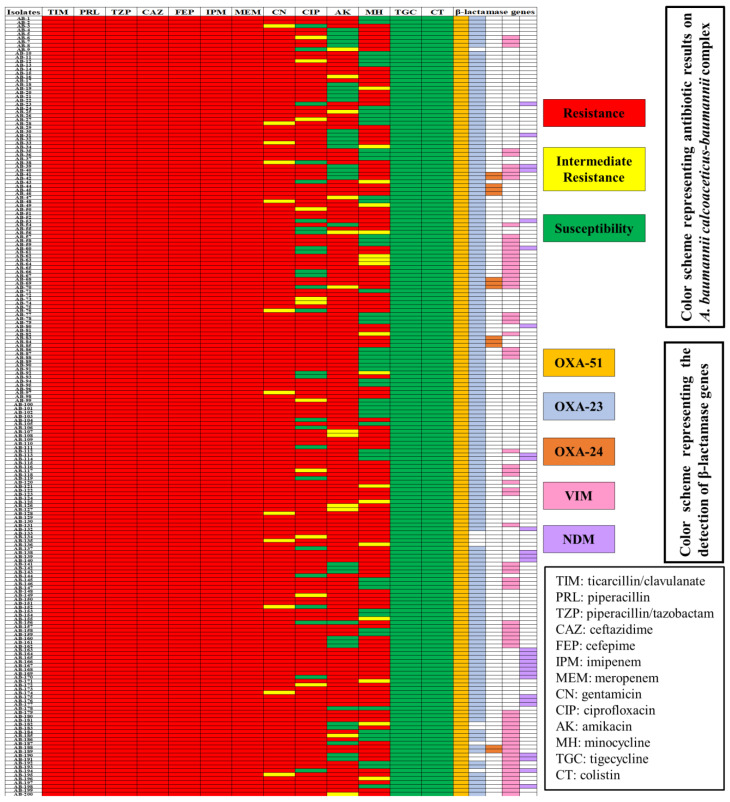
Antibiogram of CRACB isolate susceptibility to different antibiotics and the co-existence of class B and class D carbapenemase genes in the clinical isolates.

The CRACB isolates (n = 200) underwent further analysis to detect the carbapenem-resistant genes. All the isolates exhibited the intrinsic *bla*_OXA-51_ gene (100%), with high prevalence of *bla*_OXA-23_ (n = 189; 94.5%), followed by *bla*_VIM_ (n = 74; 37%), *bla*_NDM_ (n = 28; 14%), and *bla*_OXA-24_ (n = 13; 6.5%). Furthermore, the *bla*_OXA-51_, *bla*_OXA-23_, and *bla*_OXA__-24_ genes coexisted in 13 (6.5%) isolates; *bla*_OXA-51_, *bla*_OXA-23_, *bla*_OXA-24_, and *bla*_VIM_ in 7 (3.5%) isolates; and *bla*_OXA-51_, *bla*_OXA-23_, *bla*_VIM_, and *bla*_NDM-1_ in 3 (1.5%) isolates (Figure 4). The CRACB isolates with co-occurring genes displayed higher resistance than those with only a single carbapenemase gene.

## 3. Discussion

CRACB is a serious nosocomial pathogen and is the most common in critical care units around the world. Mortality and morbidity of patients with ACB are extremely high, and numerous cases of bacteremia, peritonitis, pneumonia, and urinary tract infections have been reported. ACB also causes ventilator-associated pneumonia, septicemia, and less common but significant skin, soft tissue, abdominal, and nervous system infections [15]. ACB is typically isolated from tracheal secretions and pus. In this study, 35.4% of ACB isolates were recovered from pus samples and 23.4% from tracheal secretions. Nearly identical findings were obtained in a previous study conducted in Islamabad, Pakistan, with the highest *A. baumannii* frequencies of 20% in tracheal secretions and 17% in pus samples [16]. In addition, 27.3% of *A. baumannii* isolates were obtained from pus samples in a study from Nepal [17]. However, an Indian study found a higher prevalence (64%) of *A. baumannii* in pus samples [18]. The high prevalence in pus samples might have been due to unhygienic practices and the use of unsterilized instrumentation in hospitals.

One of the most effective groups of medicines in the antimicrobial armamentarium is carbapenems, which are particularly suitable for treating multiple ACB infections. Nevertheless, increased resistance of ACB to carbapenems has been universally reported during the past decade [19]. The high level of resistance of ACB to common antibiotics is a serious concern for clinicians. In our study, 100% of the isolated bacteria were resistant to the utmost widely used β-lactams, such as cephalosporins and carbapenems (imipenem and meropenem). A study from Teheran, Iran, also showed 100% resistance to imipenem and meropenem in intensive care patients [20]. Several studies have reported the high resistance of *A. baumannii* to β-lactams, including carbapenems, in a variety of clinical settings in Pakistan [10,21,22,23]. In addition, in Bulgaria, *A. baumannii* isolates were 98.2% and 100% resistant to imipenem and meropenem, respectively [24]. A recent study of Chinese ICU patients found CRACB with significantly high resistance to tigecycline [25]; non-β-lactams (fluoroquinolones, tetracyclines, polymyxins, and aminoglycosides) were more effective against *A. baumannii* than β-lactams.

Several factors have contributed to the rise of CRACB in community clinical environments in Pakistan, such as the fragile health system, over-the-counter availability of antibiotics, the absence of microbiological diagnostic laboratories, the misuse and overuse of antibiotics, self-medication, and the socioeconomic status of patients [26,27]. The worldwide occurrence of ACB-resistant strains have further increased the concerns about this pathogen. *A. baumannii*’s high capacity to adapt, as well as its acquisition and transfer of the genetic determinants of antibiotic resistance, has rendered existing therapeutic strategies and the last range of antibiotics ineffective [28]. The acquisition and diffusion of carbapenem-resistance genes in ACB is an important factor responsible for the resurgence of resistance to last-line antibiotics. In this study, 100% of CRACB isolates were positive for *bla*_OXA-51_, 94.5% for *bla*_OXA-23_, 37% for *bla*_VIM_*,* and 14% for *bla*_NDM_. A recent study in Pakistan identified 100% of isolates with *bla*_OXA-51_ and 97% with *bla*_OXA-23_ [29], and another Pakistani study found that the *bla*_NDM_ and *bla*_OXA-23_ genes co-occurred in CRACB clinical isolates [10]. The prevalence of *bla*_OXA-23_ was found to be 100% in a study conducted in Central China [30]. In addition, two studies from China reported that 95% and 87% of *A. baumannii* isolates harbored the *bla*_OXA-23_ gene [31,32]. In previous years, fewer occurrences of *bla*_OXAs-24_ in ACB have been reported in Pakistan. However, this study found that *bla*_OXA-24_ has spread, and that it also co-exists with *bla*_OXA-23_, *bla*_OXA-51_, *bla*_VIM_, and *bla*_NDM_ in Pakistan, which implies the possibility of a similar pattern of occurrence of these genes in other less-developed countries. The high number of ACBs presenting multidrug resistance is concerning and indicates the necessity of implementing a comprehensive surveillance program for AMR. The frequent isolation of CRACB from ICUs indicates the importance of strict infection control policies in order to minimize the circulation of ACB within the ICUs and the other wards. In LMICs, patients are often accompanied by several attendants, and entry for these attendants should be restricted, particularly in ICUs. Infection risks can be reduced by frequent handwashing, wearing gloves, using disposable materials, and cleaning floors and nursing stations. The role of clinicians is pivotal in controlling the injudicious use of antibiotics. There is a need to improve the knowledge of young doctors regarding AMR in order to expand their competency in choosing the appropriate antibiotic against various multidrug-resistant bacterial strains [33].

## 4. Materials and Methods

### 4.1. Ethical Approval and Study Design 

**Figure 5:**
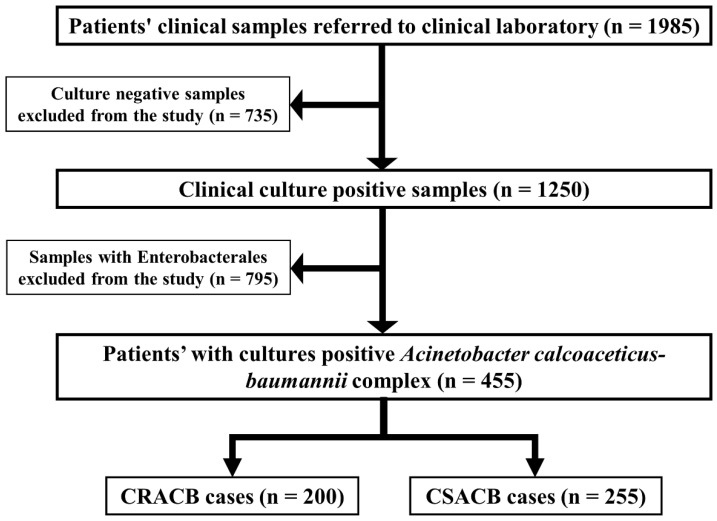
Collection of clinical samples and selection of cases with carbapenem-resistant *Acinetobacter calcoaceticus*-*baumannii* complex (CRACB) strains. Cases with carbapenem-sensitive *A. calcoaceticus*-*baumannii* complex (CSACB) were excluded from the study.

After receiving institutional approval, the study followed the Helsinki Declaration’s ethical principles [34]. In addition, the consent of all participants was obtained by assuring each that the data were used solely for research reasons and that their identity was completely concealed. This study adopted a cross-sectional analysis. A total of 1985 clinical samples from different sources (including pus, tracheal swabs, blood, cerebrospinal fluid [CSF], and urine) were referred to the laboratories of the tertiary care hospitals in Lahore and Faisalabad, Pakistan, for culture and sensitivity tests between November 2020 to May 2021. These samples were selected based on the physician’s clinical information and diagnostic judgment of suspected bacterial infection. The 200 CRACB isolates were further investigated for antibiograms and molecular characterization of antimicrobial resistant genes (ARGs) (Figure 5).

### 4.2. Case Definition

Clinical isolates were classified as carbapenem-sensitive ACB (CSACB) if susceptible to imipenem or meropenem or as CRACB if resistant or requires greater exposure to either antibiotic. Participants with at least one positive CRACB culture were considered to have CRACB. Any patient who had a positive CSACB culture was considered a CSACB case and excluded from the study.

### 4.3. Confirmation of the CRACB Phenotypic

Bacterial isolates were sub-cultured on MacConkey agar and blood agar, and the plates were aerobically incubated overnight at 37 °C. Preliminary identification was made based on colony morphology, and the isolates were confirmed using cultural characteristics and GN identification cards in an automatic VITEK 2 compact system (bioMérieux, Marcy-l’Étoile, France).

### 4.4. The Minimum Inhibitory Concentration of Antibiotics

The AMR of various antibiotics was tested against CRACB in the VITEK 2 system (bioMérieux), and the broth microdilution determined the minimum inhibitory concentrations (MICs) method. The antibiotics tested were piperacillin, piperacillin-tazobactam, ceftazidime, cefepime, meropenem, imipenem, gentamicin, tobramycin, tigecycline, minocycline, ciprofloxacin, and colistin. The MIC was interpreted per Clinical and Laboratory Standards Institute (CLSI) 2022 guidelines [35].

### 4.5. Phenotypic Confirmation of Carbapenemase 

As previously described, the modified Hodge’s test was used to confirm carbapenemase production in ACB. In summary, Hinton agar plates were inoculated with an *E. coli* strain (ATCC 25922) and a meropenem disk was placed in the middle of each plate. Clinical isolates were streaked from the disc’s edge to the entire plate and incubated overnight at 37 °C [36]. A clover indentation was considered a positive result. The presence of carbapenemases was further confirmed using a more recent modified carbapenem inactivation (mCIM) method and was interpreted in accordance with the CLSI guidelines [35]. The isolates were well-preserved at −82 °C in Luria Bertani broth, which contained 14% glycerol.

### 4.6. Molecular Identification of Carbapenemase Genes

DNA extraction was carried out by the boiling method prior to amplification. All submitted ARG determinants were augmented by nested PCR using sequence-specific primers [37]. The primers that were used in the study are listed in Table 3. The initiation temperature was 94 °C for 5 min, followed by a denaturation temperature of 94 °C for 30 s. Annealing was carried out at 52 °C for 40 s, followed by a final extension at 72 °C for 6 min [38]. Lastly, the amplified fragments were separated according to the molecular weight/size of the base pair and compared to a DNA scale of 100 bps. Fragments were displayed under UV light using a gel documentation system (Bio-Rad, Watford, UK).

### 4.7. Data Analysis

The IBM SPSS v.26 (IBM, Chicago, United States) and GraphPad Prism 9.0.1 (GraphPad Software, Inc., San Diego, United States) were used for data analysis. The variables were analyzed using descriptive statistics, and a chi-square test was done to calculate *p*-values. In this study, a *p*-value less than 0.05 was considered significant. 

## 5. Conclusions

The detection of several CRACB in our study is a substantial public health concern. This study found that this highly critical pathogen was resistant to almost all categories of AWaRe antibiotics, except tigecycline and colistin. The *bla*_OXA-23_, *bla*_OXA-24_, *bla*_NDM_, *bla*_OXA-51_ and *bla*_VIM_ genes were found to co-occur in some CRACB isolates. The spread of these pathogens nationally and internationally raises serious concerns; therefore, an active and effective national AMR surveillance study should be conducted, and the Ministry of Health of Pakistan should implement the National Action Plan on AMR. The use of collective strategies, such as improving cleaning processes, utilizing aseptic techniques, disposing of old furniture, controlling hospital visitors, and applying stringent policies on antibiotic use, may contribute to the elimination of CRACB.

## Figures and Tables

**Table 1:** Clinical characteristics and patient demographics of CRACB and CSACB cases.

Clinical Characteristic	CRACB (n = 200)		CSACB (n = 255)		*p*-Value
	n	%	n	%	
**Age (Years)**					
Range	1–95		1–87		
Mean (±standard deviation)	43.5 ± 17.5		46.8 ± 19.2		0.05
**Gender**					
Male	112	56	152	59.6	0.43
Female	88	44	103	40.3	
Male to female ratio	1.27: 1		1.47:1		
**Clinical Sample**					
Pus	79	35.4	102	40.9	0.91
Tracheal secretions	47	23.5	59	23.1	0.92
Sputum	21	10.5	33	12.9	0.42
Blood	20	10	27	10.5	0.83
CSF	17	8.5	11	4.3	0.46
Urine	11	5.5	16	6.2	0.72
Bile fluid	5	2.5	7	2.7	0.87
**Hospital Ward**					
ICU	77	38.5	93	36.4	0.65
MWW	37	18.5	46	18	0.89
FMW	36	18	41	16	0.58
CCU	30	15	39	15.2	0.93
OPD	12	6	21	8.2	0.36
OT	8	4	15	5.8	0.34

CSF: cerebrospinal fluid; ICU: intensive care unit; MMW: male medical ward; FMW: female medical ward; CCU: critical care unit; OPD: outpatient department; OT: operation theater.

**Table 2:** Dissemination of CRACB from clinical sources and hospital wards.

Clinical Source	Hospital Ward					
	ICU	MMW	FMW	CCU	OPD	OT
Pus (n = 79; 39.5%)	31 (49.3%)	16 (20.2%)	12 (15%)	12 (15%)	7 (8.8%)	1 (7.9%)
Tracheal secretions (n = 47; 23.5%)	19 (40.4%)	7 (14.8%)	11 (23.4%)	8 (17%)	2 (4.2%)	-
Sputum (n = 21; 10.5%)	6 (28.5%)	7 (33.3%)	-	2 (9.5%)	1 (4.7%)	5 (23.8%)
Blood (n = 20; 10%)	12 (60%)	-	1 (5%)	5 (25%)	-	2 (10%)
CSF (n = 17; 8.5%)	3 (17.6%)	3 (17.6%)	8 (47%)	2 (11.7%)	1 (5.8%)	-
Urine (n = 11; 5.5%)	3 (27.2%)	4 (36.3%)	2 (18%)	-	2 (18%)	-
Bile fluid (n = 5; 2.5%)	3 (60%)	1 (20%)	1 (20%)	-	-	-

CSF: cerebrospinal fluid; ICU: intensive care unit; MMW: male medical ward; FMW: female medical ward; CCU: critical care unit; OPD: outpatient department; OT: operation theater.

**Table 3:** The list of primers used in the study.

Target Gene	Primer Sequence (5′-3′)	Size (bp)
*bla*_OXA23-F_	GATCGGATTGGAGAACCAGA	501
*bla*_OXA23-R_	ATTTCTGACCGCATTTCCAT	
*bla*_OXA24-F_	GGTTAGTTGGCCCCCTTAAA	246
*bla*_OXA24-R_	AGTTGAGCGAAAAGGGGATT	
*bla*_OXA51-F_	TAATGCTTTGATCGGCCTTG	353
*bla*_OXA51-R_	TGGATTGCACTTCATCTTGG	
*bla*_VIM-F_	GATGGTGTTTGGTCGCATAA	390
*bla*_VIM-R_	CGAATGCGCAGCACCA	
*bla*_NDM-F_	GGTTTGGCGATCTGGTTTTC	699
*bla*_NDM-R_	CGGAATGGCTCATCACGATC	

## Data Availability

The data presented in this study are available on request from the corresponding authors.
